# Supplementary material for: PLOS Neglected Tropical Diseases 2015 Reviewer Thank You
Source: PLoS Negl Trop Dis. 2016 Feb 23;10(2):e0004520. doi: 10.1371/journal.pntd.0004520 (PMC4764366; doi:10.1371/journal.pntd.0004520)
Supplement: S1 Reviewer List — (PDF) [file pntd.0004520.s001.pdf]

PLOS | NEGLECTED TROPICAL DISEASES

PLOS NTDs would like to thank all those who reviewed on behalf of the journal in 2015:

John Aaskov
Fernando Abad-Franch
Emmanuel Abatih
Jessica Abbate
Mohamed Abdel Hakeem
Nada Abdelmagid
Bernadette Abela-Ridder
Bayeh Abera
Judith Aberle
Anthony Ablordey
David Abraham
Jônatas Abrahão
Ali Acar
Nicole Achee
Alvaro Acosta-Serrano
Rodney Adam
L. Adams
Emily Adams
Linda Adams
David Adamson
Vanessa Adaui Sicheri
David Addiss
Ayola Adegnika
Zack Adelman
Ifedayo Adetifa
Babatunde Adewale
Bipin Adhikari
Philip Adongo
Vincent Adung'A
Toni Aebischer
Luis Afonso
Farhat Afrin
Suneth Agampodi
Madhur Aggithaya
Ellen Agler
Philip Agnew
George Agogo
Rodrigo Aguiar Soares
Tile Ah Leong
Muhamad Rusdi Ahmad
Rusmili
Kamruddin Ahmed
Steven Aird
Wataru Akahata
Ajoke Akinola
Oladele Akogun
Jean Paul Akue
Kalichamy Alagarasu
Alejandro Alagon
Jordi Alberola
Audrey Albertini
Marco Albonico
Letusa Albrecht
Jared Aldstadt
Abebe Alemu
James Alexander
Neal Alexander
Kathleen Alexander
Nahid Ali
Amer Al-Jawabreh
Mohan Alladi
Jean-Pierre Allain
Kathryn Allan
Rachel Allavena
Denise Allen
Igor Almeida
Rodrigo Almeida-Paes
Luke Alphey
Luke Alphy
Khaled M. Al-Qaoud
J. Alspaugh
Julio Altamirano
Benjamin Althouse
Karl-Heinz Altmann
Daniel Altmann
Kishore Alugupalli
Fernando Alvarez-Valin
Ananda Amarasinghe
Maria Lourdes Amarrillo
Valdir Amato
Javier Ambrosio
Nana Ama Amissah
Yaw Amoako
Emmanuel Ampofo
Julia Ampuero
Kathryn Anderson
Luciana Andrade
Marcos Andre
Jason Andrews
Germán Añez
Andrea Angheben
Andrey Anisimov
Serge Ankri
Nasim Ansari
Rashid Ansumana
Carlos Antunes
Ruslan Aphasizhev
Charles Apperson
Byron Arana
Minerva Arce
Ana Paula Arez
Larry Arlian
Naomi Aronson
Sridhar Arumugam
Hilmir Asgeirsson
Joseph Ashour
Anthony Ashton
Margaret Astin
Katherine Atkins
Louise Atkinson
Geoffrey Attardo
Sarah Auburn
Vicky Avery
Guillermina Avila
Tatjana Avšic-Županc
C.F.J. Ayres
Abdu Azad
Kifaya Azmi
Simon Babayan
Olivia Bacellar
Roberto Badaró
Karoun Bagamian
Robin Bailey
Florence Baingana
J. Baird
Stephen Baker
Tamas Bakonyi
Nandhakumar Balakrishnan
Mangai Balasegaram

Fabrizio Balestrino
Guy Ballard
Gad Baneth
Charles Bangham
James Bangs
Melanie Bannister-Tyrell
Ashley Banyard
Changjun Bao
Clara Barbiéri
Alan Barbour
Corentin Barbu
Christovam Barcellos
Beatrice Barda
Elisa Baring
Celine Barnadas
Yves Barogui
John Barr
Simon Barratt-Boyes
Roberto Barrera
Janine Barrett
Kim Barrett
Michael Barrett
Alan Barrett
Roberto Barros
Eileen Barry
Alyssa Barry
Daniella Bartholomeu
Anirban Basu
Arnaud Bataille
Imelda Bates
Paul Bates
Jake Baum
Dennis Baumgardner
Sina Bavari
Abdulhakim Bawadekji
Matthew Baylis
Chiara Bazzocchi
Charles Beard
David Beasley
Lynette Beattie
P. Beatty
Mark Beatty
Ingeborg Becker
Sören Becker
Nigel Beebe
James Beeson
Susanta Behura
John Beier
Vicente Belizario
Anita Bell
Silvana Belo
Gil Benard
M. Benbow
Mark Benbow
Scott Bender
Malcolm Bennett
Sasisekhar Bennuru
Joshua Benoit
Dennis Bente
Franck Berger
Eric Bergeron
Luiz Bermudez
Caryn Bern
Kristen Bernard
Sonja Best
Bernard Bett
Stephen Beverley
Tariku Beyene
Suvendra Bhattacharyya
Quentin Bickle
Franziska Bieri
Michael E. Bigby
James Bina
Henry Binder
Mike Bingham
Brian Bird
Nana Biritwum
Gerd Birkenmeier
David Bishai
Zeno Bisoffi
William Black Iv
Jason Blackburn
Stuart Blacksell
Carol Blair
David Blair
Karl Blanchet
Jesse Blanton
Lucas Blanton
Jon Blevins
James Bliska
Bradley Blitvich
Melanie Blokesch
Marshall Bloom
Maria Heloísa Blotta
Brittany Blouin
Jamie Blow
Johannes Blum
Daniel Boakye
Boakye Boatin
Ladaporn Bodhidatta
Marleen Boelaert
Jonathan Bohbot
Pascal Boireau
Matthew Bonds
Bassirou Bonfoh
Marcelo Bonini
Mariangela Bonizzoni
Sarah Bonnet
Alison Booth
Mark Booth
Jeff Borchert
Valeria Borges
Calderon Boris
Piotr Borkowski
Silvia Boscardin
Michael Boshart
Kwabena Bosompem
Monica Botelho
Emmanuel Bottieau
Christian Bottomley
David Boulware
Claire Bourke
Kostas Bourtzis
Michel Boussinesq
Donald Bouyer
Jérémy Bouyer
Saeid Bouzari
Asha Bowen
Leigh Bowman
Ross Boyce
Marcelo Bozza
Doug Brackney
David Bradley
David Bradley
Molly Brady
Oliver Brady
Cynthia Braga
Sara Brant
Pierre Brantus
Allan Brasier
Patrícia Brasil
Martin Bratschi
Aaron Brault
Lulu Bravo

Reginaldo Brazil
Klaus Brehm
David Brett-Major
Laurence Briant
Seth Britch
Carlos Brito
Constança Britto
Warwick Britton
Cláudia Brodskyn
William Brogdon
Simon Brooker
Roland Brosch
Hannah Brown
Heidi Brown
Katherine Brown
Reto Brun
Fabrizio Bruschi
Randy Brutkiewicz
Laurent Brutus
A.D.M. Bryceson
Amy Buck
Frederick Buckner
Philip Budge
Christine M. Budke
Pierre Buekens
Pierre Buffet
Salome Bukachi
Jesse Bump
Stewart Burgess
Tristan Burgess
Felicity Burt
Mary Burtnick
Sakib Burza
Carlos Buscaglia
Philippe Büscher
Florence Buseyne
Philippe Busso
Dulce Bustamante
Juan Bustamante
James Butler
Noah Butler
Klaus Buttenschoen
Brian Byrd
Ana Caceres
Conor Caffrey
Tina Calderon
Carlos Eduardo Calzavara-Silva

Tamara Camara
Emmanuelle Cambau
Caroline Cameron
Suzanne Campbell
Lindsay Campbell
Oscar Campetella
Renata Candido
Ermanno Candolfi
Luz Cano
Paul Cantey
Margareth Capurro
Hélène Carabin
Eric Cardinale
Luis Cardoso
Graeff-Teixeira Carlos
Jane Carlton
Elizabeth Carlton
David Carmena
Cláudia Carneiro
Jason Carnes
Simon Carpenter
Arturo Carpio
Vanessa Carregaro
K. C. Carter
Nicola Carter
Ben Carter
Lucas Carvalho
Edgar Carvalho
Tecia Carvalho
Nicholas Casewell
Benjamin Cash
Maria Cassera
Léa Castellucci
Maria Castillo
Rodolfo Castro
William Castro-Borges
Adriano Casulli
Clayton Caswell
Flaminia Catteruccia
Marta Cavalcanti
Bernard Cazelles
Maria Cecere
Arturo Centurion-Lara
Carla Cerami
Hugo Cerecetto
Manuel Cespedes
Hee-Jae Cha
Jose Chabalgoity

Kris Chadee
Dave Chadee
Jong-Yil Chai
Wanpen Chaicumpa
Amit Chakraborty
Frédérique Chammartin
Donald Champagne
James Angus Chandler
Yung-Fu Chang
Li-Yen Chang
Narisara Chantratita
Dennis Chao
Chien-Chung Chao
Day-Yu Chao
François Chappuis
Theeraphap Chareonviriyaphap
Remi Charrel
Eric Chatelain
Mitali Chatterjee
Amitabha Chattopadhyay
Ian Cheeseman
Xi Chen
Shi Chen
Qin Cheng
Bobby Cherayil
Harrell Chesson
Christophe Chevillard
Roma Chilengi
Neil Chilton
Richard Chipman
Nakul Chitnis
Lars Chittka
Nam-Hyuk Cho
Bruno Chomel
Ashok Chopra
Gerardo Chowell
Justin Jang Hann Chu
Candy Chuah
Ampaiwan Chuansumrit
Federico Cicora
Ruben Cimino
Alexander Ciota
Hannah Clapham
Thomas F. Clasen
Christine Clayton
Sarah Cleaveland
Archie Clements

Julie Clennon
Jan Clerinx
Gilles Clermont
Florence Cliquet
Axel Cloeckaert
Jenifer Coburn
Claudia Codeço
Flavio Coelho
Eduardo Coelho
Luc Coffeng
Lark Coffey
Christian Coles
James Collins Iii
Gonzalo Colmenarejo
Tonya Colpitts
Iñaki Comas
Marcelo Comini
Eric Comte
Fatima Conceição-Silva
Jan Conn
Bernadette Connolly
John Connor
Paul Converse
David Conway
Joseph Cook
Philip Cooper
Isabelle Coppens
Anabela Cordeiro-Da-Silva
Martin Cormican
Cynthia Cornelissen
Emmanuel Cornillot
Simon Corrie
Betina Corsico
Paul Corstjens
Sofia Cortes
Mauro Javier Cortez Veliz
Andrew Corwin
Carlos Costa
Gláucia Cota
Jean Coulibaly
José Coura
Orin Courtenay
Paul Courtright
Christine Coustau
Iliano Coutinho-Abreu
Bernard Couvreur
Benjamin Cowling
Jonathan Cox
Keren Cox-Witton
Christina Coyle
Thomas Crellen
Julio Croda
Simon Croft
Peter Crompton
Thomas Cropper
Robert Cross
Kathryn Crowley
Adriana Cruz
Liwang Cui
Richard Culleton
Narcisa Cunha-E-Silva
Edecio Cunha-Neto
Adam Cunningham
Eddie Cupp
Jeffrey Currier
Sally Cutler
Krystyna Cwiklinski
Clarissa Da Costa
Joao Da Rosa
A. Menezes Da Silva
João Da Silva
Valerie D'Acremont
Alda Maria Dacruz
Johanna Daily
John Dalton
John Dalton
Emilie Dama
Stefano D'Amelio
María Damiani
David Dance
Rachel Daniels
Anthony Danso-Appiah
F. Danson
Filipe Dantas-Torres
Jonathan Darbro
Bernard Dardzinski
Jai Das
Pradeep Das
Gregory Dasch
Alexandre Dasilva
Virginia Dato
Claudia Daubenberger
Regina Daumas
Miles Davenport
Robert Davey
Dan David
William Davis
Egon Daxbacher
Nicholas Day
Tim Day
Marcos De Almeida
William De Glanville
Sybren De Hoog
Vinicio De Jesus Perez
Bouke De Jong
Sanne De Jong
Harry De Koning
José De La Fuente
John De La Parra
Isabel De Miranda Santos
Rosimeire De Oliveira
Camila De Oliveira
Aravinda De Silva
Nilanthi De Silva
Jose De Sousa-Figueiredo
Wanderley De Souza
Sake De Vlas
Henry De Vries
Deborah Dean
Keith Dear
Alexander Debrah
Chitrita Debroy
Saskia Decuypere
George Deepe, Jr.
Gabriel Defang
Rosa Maria Del Angel
Oscar Del Brutto
Maurizio Del Poeta
Hernando Del Portillo
Ricardo Demarco
Ana Denicola
David Denning
Paul Denny
Jérôme Depaquit
Peter Deplazes
Kebede Deribe
Albert Descoteaux
David Deshazer
Harendra Desilva
Philippe Desprès
Eileen Devaney
Shamala Devi
Gregor Devine
Angela Devine

Ranadhir Dey
Dharumadurai Dhanasekaran
Radhika Dhingra
Roberto Di Santo
Mawlouth Diallo
Diawo Diallo
Alvaro Diaz
Sean Diehl
David Diemert
Ralf Dietzgen
Peter Diggle
Arie Dijkstra
Nedialko Dimitrov
Rhoel Dinglasan
Trung Dinh The
Lileia Diotaiuti
Ermias Diro
Colette Dissous
Katharina Dittmar
Maria Diuk-Wasser
Rubens Do Monte-Neto
Carlota Dobano
Stephen Dobson
Roberto Docampo
Kimberly Dodd
Mike Doenhoff
Ana Domingos
Sheila Donnelly
Patricia Dorn
Marcus Dorner
Eric D'Ortenzio
Tania Dottorini
Richard Douglass
Ogobara Doumbo
Steven Dow
Kimberly Dowd
Jennifer Downs
Philip Downs
Tom Drake
Christopher Drakeley
Michael Drebot
Guangcai Duan
Jean Dubuisson
Frederic Ducancel
J. Stephen Dumler
Stephen Dumler
Eric Dumonteil
Robert Duncan
Sandra Durães
Anna Durbin
Salome Dürr
Jonathan Dushoff
Michael Duszenko
Walderez Dutra
Jan Dvorak
Vit Dvorak
Julian Eaton
Gregory Ebel
Mark Eberhard
Hideki Ebihara
Johannes Eble
Michael Eddleston
Miriam Eddyani
Robert Edelman
John Edman
Sophie Edouard
Michael Efunshile
Rosalind Eggo
Andrea Egizi
Uwem Ekpo
Mahmoud Elhaig
Maria Carolina Elias
Celina Elissondo
Alison Elliott
Esther Ellis
Brett Ellis
James Ellison
Michael Emch
Nancy Endersby
Timothy Endy
Markus Engstler
Christian Engwerda
Delia Enria
Jeroen Ensink
Jonathan Epstein
Marina Eremeeva
Koray Ergunay
Kacey Ernst
Hildegund Ertl
Eugene Erulu
Ananias Escalante
Luis Escobar
Galileo Escobedo
Ana Espino
Avelina Espinosa
Bertha Espinoza
Maria Cristina Espírito-Santo
Socorro Espuelas
Danuza Esquenazi
José Esteban Sanchis
J Esterhuizen
Magnus Evander
Tom Evans
Amara Ezeamama
Ahmed Fahal
Anna-Bella Failloux
Roy Faiman
Geoffrey Fairchild
Alan Fairlamb
M. Abul Faiz
Franco Falcone
Padraic Fallon
Ferric Fang
Yuan Fang
Liqun Fang
Chuck Farah
Ary Faraji
Nuno Faria
Mac Farnham
Nicolas Fasel
Vinicius Fava
Amani Fawzi
Nicholas Feasey
Margaret Feeney
David Fegan
Christine Fehlner-Gardiner
Andrew Fei
Mohammad Mehdi
Feizabadi
Heinz Feldmann
Hermann Feldmeier
Alan Fenwick
Michael Ferguson
Maria Cecilia Fernandes
Stefan Fernandez
Ana Fernandez-Sesma
Pedro Fernández-Soto
Lakkumar Fernando
Rafaela Ferreira
Marcelo Ferreira
Ana Ferreira
Arturo Ferreira
Eric Fevre
Mark Field

Luisa Figueiredo
Laurent Filleul
Paul Fine
Katja Fink
Alireza Firooz
Egil Fischer
Peter Fischer
Marc Fischer
Anne Fischer
Katja Fischer
William Fischer
David Fisman
Christopher Fitzpatrick
Bernhard Fleischer
Fiona Fleming
Agnes Fleury
Ana Flisser
Lucile Floeter-Winter
Adriana Flores
Robin Flynn
Grace Fobi
Lane Foil
Desmond Foley
Janet Foley
Dina Fonseca
Cristina Fonseca
Anthony Fooks
Naomi Forrester
Brett Forshey
Anny Fortin
Allen Foster
Florence Fouque
Tatiana Fraga
Deborah Fraga
Jose R. Franco
Octávio Franco
Carlos Franco-Paredes
Nikolaos Frangogiannis
Richard Franka
Alexander Franz
Matthew Freeman
Alexander Freiberg
Hector Freilij
Michael French
Francesca Frentiu
Manuel Fresno
Ute Frevert
Michal Fried
David Friedman
Jennifer Friedman
Elena Frolova
Ricardo Fujiwara
Claire Fuller
Robert Fuller
Isaac Chun-Hai Fung
João M Furtado
Janet Fyfe
Paolo Gabrieli
Albis-Francesco Gabrielli
Kenneth Gage
Kenneth Gage
Hernando Gaitán-Duarte
Jim Gallarda
Kerry Gallo
Luca Galluzzi
Alison Galvani
Francisco Gamarro
Manoj Gambhir
Yunn-Hwen Gan
Ekambaram Ganapathy
Jean-Pierre Gangneux
Nirmal Kumar Ganguly
Sreenivas Gannavaram
Tomas Ganz
Xin Gao
Teresa Garate
Amadou Garba
Leandro Garcia
Paul Garner
Robert Garry
Joaquim Gascon
Philippe Gasque
Katherine Gass
Robin Gasser
Michael Gaunt
Gregory Gauthier
Philippe Gautret
Bernard Alex Gauzere
Cesar Gavidia
Indika Gawarammana
Nicholas Gay
Charlotte Gaydos
Bruce Gaynor
Timothy Geary
Lashitew Gedamu
Jay Gee
Vincent Geenen
Stanny Geerts
Florian Gehre
Stefan Geiger
Brian Geiss
Moti Yohannes Gemechu
Claudio Genchi
Nicholas Generous
Patrick Gérardin
Bruno Ghersi
Aniruddha Ghose
Lorenzo Giacani
Wendy Gibson
Amy Gilbert
John Gimnig
Howard Ginsberg
Federica Giordani
Ricardo Giordano
Emanuele Giorgi
Patrick Giraudoux
Núria Gironès
John Githure
Rodolfo Giunchetti
Michelle Gleason
Lucy Glover
Cyrille Goarant
Edessa Gobena
Geoffrey Gobert
Tony Goldberg
Allison Golden
Kenneth Gollob
Estelio Gomberg
Gabriela Gomes
Juliana Assis Gomes
Beatriz Gomez
Hernando Gomez
Karina Gomez
Nelder Gontijo
Armando Gonzalez
John González
Camila González
Dolores González-Pacanowska
Ian Goodhead
Rodion Gorchakov
Catherine Gordon
Aubree Gordon
H. Ulrich Göringer

Marga Goris
David Gorla
Christian Gortazar
Jean-Pierre Gorvel
Roland Gosling
Hiro Goto
Nicole Gottdenker
Bruno Gottstein
Ernest Gould
Fred Gould
Sebastien Gourbiere
Brian Gowen
Emily Gower
Dennis Grab
Luigi Gradoni
Brian Graham
Stewart Graham
Marina Gramiccia
Warwick Grant
Raoul Grasman
Stephen Graves
Patricia Graves
Darren Gray
Jeremy Gray
Sharone Green
Robert Greenberg
Richard Greenberg
Anthony Gregory
Aric Gregson
Elli Greisenegger
Christoph Grevelding
Mario Grijalva
Maria Eugenia Grillet
Brian Grimberg
Laura Grogan
Max Grogl
Uwe Gross
Jacques Grosset
Alexandra Grote
Christoph Grunau
Duane Gubler
Patrick Guerin
Humberto Guerra
Marta Guerra
Richard Guerrant
Gilles Guerrier
Claudia Guezala
Felipe Guhl
Nancy Guillen
Lamia Guizani-Tabbane
Abba Gumel
David Gurarie
Greg Gurri-Glass
Ricardo Gürtler
José Gutiérrez
Theresa Gyorkos
David Haake
Abdulrazaq Habib
Georg Häcker
Danny Haddad
Jose Hagan
Ferry Hagen
Rosane Hahn
Asrat Hailu
Lee Haines
Robert Hall
Roy Hall
Christopher Hall
Jo Halliday
Eric Halsey
Scott Halstead
Shinjiro Hamano
Omar Hamarsheh
Nicholas Hamm
Katie Hampson
Penelope Hancock
Kathryn Hanley
Kimberly Hanson
John Hargrove
David Harley
Donald Harn
Billy Harnett
Laura Harrington
Jason Harris
John Harris
Robert Harrison
Susanne Hartmann
Futoshi Hasebe
Epco Hasker
Jan Hattendorf
Susanna Hausmann-Muela
Bill Hawley
Thomas Hawn
Roderick Hay
Tetsuya Hayashi
Mary Hayden
David Heath
Miriam Heijnders
Robert Heinzen
Mark Heise
Joseph Heitman
Trina Helderman
Helena Helmby
Andrés Henao-Martínez
Sarah Hendrickx
David Hendrickx
Lars Henning
Andrea Henriques-Pons
Maria Herrera
Manuel Hetzel
Jorg Heukelbach
Roger Hewson
Peter Heydemann
Alicia Hidron
Tarig Higazi
Markus Hilty
B. Joseph Hinnebusch
Jay Hinton
Alec Hirsch
Hajime Hisaeda
Eric Hoberg
Natasha Hochberg
Mary Hodges
Achim Hoerauf
Anders Hofer
Cornelis Hokke
Wim Hol
Michael Holbrook
Celia Holland
Martin Holland
Edward Holmes
Kristin Holt
Deborah Holt
Mamoun Homeida
Robert Hontz
Adrian Hopkins
Carol Hopkins Sibley
Antje Hoppenheit
Tiffany Horng
Michael Horseman
Olaf Horstick
John Horton
Parviez Hosseini
Peter Hotez

S.L. Hoti
Eric Houpt
Peter Howe
Michael Hsieh
Yi-Ju Hsieh
Wei-Cheng Hsu
Wei Hu
Guihua Huang
Yan Huang
Zdenek Hubalek
Marc Hübner
Grant Hughes
Raymond Hui
Judith Humphries
Debbie Humphries
Elizabeth Hunsperger
Eveline Hürlimann
Ivy Hurwitz
Kris Huygen
Jenny Hyde
Noreen Hynes
Atila Iamarino
Ikuo Igarashi
Tetsuro Ikegami
Nicola Illing
Turgut Imir
Noboru Inoue
Dennis Ip
Lourdes Isaac
Alison Isaacs
Geoffrey Isbister
Nahed Ismail
Luis Izquierdo
Shuji Izumo
Ricardo Izurieta
Inuwa Jaafaru
Alan Jackson
Joseph Jackson
Andrew Jackson
Alan Jackson
Yves Jackson
Robert Jacobs
Jan Jacobs
Frederique Jacquerioz
Thomas Jaenisch
Charles Jaffe
Ana Jansen
Petrus Jansen Van Vuren
Richard Jarman
Douglas Jasmer
Emilie Javelle
Graham Jenkin
Stephen Jenkins
David Jenkins
Claire Jenkins
Helen Jenkins
Selma Jeronimo
Aaron Jex
Tariku Jibat
Maria Isabel Jimenez
Anja Joachim
Maria Johansen
Michael Johansson
Jacob John
Perfect John
Annamma John
Barbara W. Johnson
Tammi Johnson
Paul Johnson
Lucinda Johnson
Emmitt Jolly
Douglas Jones
Christopher Jones
Malcolm Jones
Rey Jorge
Peter Jourdan
Justin Julander
Jonathan Juliano
Thomas Junghanss
Caroline Junqueira
Inacio Junqueira De Azevedo
Antarpreet Jutla
Narcis Kabatereine
Malika Kachani
Rebekah Kading
Stuart Kahn
Christoph Kaiser
Maria Kaiser
Laurent Kaiser
Siripen Kalayanarooj
Shiv Kale
Andre Kalil
Khumbo Kalua
Joseph Kamgno
Shaden Kamhawi
Edna Kaneshiro
Gagandeep Kang
Anu Kantele
Anu Kantele
Durrell Kapan
Stephen Kaptoge
Ece Karatan
Shahid Karim
Samuel Kariuki
Hiroaki Kariwa
Maria Karlsson
Ajit Karna
Harin Karunajeewa
Nadira Karunaweera
Shinji Kasai
Fatah Kashanchi
Randee Kastner
Anuradhani Kasturirathna
Anuradhani Kasturiratne
Francis Xavier Kasujja
Moses Katabarwa
Shin-Ichiro Kawazu
Rudovick Kazwala
Marguerite (Kelly) Keating
M. Keckler
Karen Keddy
Jeremy Keenan
Martine Keenan
Jennifer Keiser
Jennifer Keller
Paul Kelly
John Kelly
Ben Kelly
Louise Kelly-Hope
Kemparaju Kempaiah
Volkhard Kempf
Leo Kenefic
Malcolm Kennedy
Ernest Kenu
Peter Kern
Ligia Kerr
Natkunam Ketheesan
Nemat Keyhani
Julius Keyyu
Abdelouahed Khalil
Ali Khamesipour
Rajiv Khandekar
Alireza Khatami
Moran Ki

Rudo Kieft
Mariana Kikuti
A. Kilpatrick
Young Eun Kim
Peter Kima
Akinori Kimura
Jonathan King
Nicholas King
Douglas Kinghorn
Robert Kingsley
Safari Kinung'Hi
Martyn Kirk
Theo Kirkland
Jessica Kissinger
Patricia Kissinger
Paul Klatser
Thomas Klei
Terry Klein
Michele Klingbeil
Sandor Klis
Karl Klose
Gerhart Knerer
Charles Knirsch
Darryn Knobel
Stefanie Knopp
Oliver Koch
Constantianus Koenraadt
Cristian Koepfli
Nobuo Koizumi
Vijaya Kolachalama
Nicholas Komar
Yoon Kong
Sukhontha Kongsin
Dimitrios Kontoyiannis
Wojciech Kopec
Joseph Koroma
Gülay Korukluoglu
Donna Koslowsky
Michael Kosoy
Michalis Kotsyfakis
Andrew Kotze
Benjamin Koudou
Artemis Koukounari
Athena Kourtis
Georgios Koutsovoulos
Uriel Koziol
Elliot Krafsur
Peter Kraiczy
Peter Krause
Alison Krentel
Marco Krieger
Manoj Krishnan
Igor Krizaj
Axel Kroeger
Alejandro Krolewiecki
Jürgen Krücken
Detlev Krüger
Girish Ks
Ulrich Kuch
Richard Kuhn
Senanayake Kularatne
Daniel Kulke
N. P. Kumar
Sanjai Kumar
Jonathan Kurtis
Sanjaya Kuruppu
Ivan Kuzmin
Babu L. Tekwani
Marcus Lacerda
Juan Laclette
William Lafuse
Heimo Lagler
Ren Lai
Benoit Laleu
E. Laloy
Phung Lam
Hanh Lam
Saba Lambert
Poppy Lamberton
Louis Lambrechts
Patrick Lammie
Claudio Lanata
Scott Landfear
Sandra Laney
Jean Lang
Gordon Langsley
Felix Lankester
Joseli Lannes-Vieira
Carlos Lanusse
Kamolwish
Laoprasopwattana
Dhafer Laouini
Renee Larocque
Vicente Larraga
Sébastien Larréché
Daniel Larremore
Tamás Laskay
Colleen Lau
Marcia Laurenti
Francisco Laurindo
Mallika Lavania
James Lawler
Rachel Lawrence
Scott Lawton
Phillip Lawyer
Laura Layland
Helen Lazear
Claudio Lazzari
Arnaud Le Menach
Clint Leach
Herve Lecoeur
Marc Lecuit
Luis Lecuona
Bruce Lee
Min-Shi Lee
Rosemary Lees
Rosemary S. Lees
Pedro Legua
Veerle Lejon
Tiziana Lembo
Audrey Lenhart
Suzanne Lenhart
Deborah Lenschow
Yee-Sin Leo
Guillermo Leon
Isabelle Leparc-Goffart
Norm Leppla
Andres Lescano
Francois Xavier Lescure
Bruno Levecke
Paul Levett
Michael Levin
Myron Levine
Michael Levy
Lisa Lewis
Michael Lewis
Joseph Lewnard
Jun Li
Chunhao Li
Joseph Liao
Daniel Libraty
Tore Lier
Marc Liesa
Bernhard Liese

Marshal Lightowlers
Walter Lilenbaum
Pharath Limmathurosakul
Direk Limmathurosakul
Renyong Lin
Hualiang Lin
José Angelo Lindoso
Qiyong Liu
Jingze Liu
Haizhou Liu
Jun Liu
Weibin Liu
Song Liu
Desheng Liu
Alejandro Llanos
Martin Llewellyn
Alun Lloyd
Yi-Chun Lo
Shawn Lockhart
Diana Lockwood
James Logan
Philipe Loiseau
P'Ng Loke
Bruno Lomonte
Kanya Long
Marcela Lopes
Benedito Lopes da Fonseca
Job Lopez
Rogelio Lopez-Velez
Sandra Lopez-Verges
Jacob Lorenzo-Morales
Christine Loscher
Hannelore Lotter
Valérie Louis
Alex Loukas
José Lourenço
Flávio Loures
Hechmi Louzir
Philip Loverde
Jenny Low
Warren Lowman
Elizabeth Loza-Rubio
Stephen Luby
Paula Ludovico
Helena Lugão
Igor Lukashevich
Sheila Lukehart
Julius Lukes
Lucy Lum
Zhao-Rong Lun
Jennifer Lund
Francisco Luquero
Lalwo Luroni
Sara Lustigman
Paula Luz
Kleber Luz
Shan Lv
G. Marshall Lyon
Musawenkosi Mabaso
David Mabey
Kevin Macaluso
Andrew MacDonald
Paulo Roberto Machado
Fabiana Machado
Charles Mackenzie
Jason Mackenzie
Stephen Mackessy
Calman MacLennan
Annette MacLeod
Alastair MacMillan
Purnima Madhivanan
Henry Madsen
Fabrizio Mafessoni
Stephen Magesa
Stefan Magez
Alan J. Magill
Jean-Francois Magnaval
James Maguire
Suresh Mahalingam
Siddhartha Mahanty
Rajendra Maharaj
Radha Maheshwari
Barbara Mahon
Carla Maia
Luigi Maiorano
Phelix Majiwa
Subrata Majumdar
Benjamin Makepeace
Laurence Malandrin
Gathsaurie Malavige
Paritosh Malaviya
Claudius Malerczyk
David Malone
John Malone
Mark Manary
Chitra Mandal
Laura Manna
Patricio Manque
Pablo C. Manrique Saide
Pablo Manrique-Saide
Ben Mans
Daniel Mansur
Pablo Maravilla
Matteo Marcantonio
Paula Marcet
Laurence Marchat
Richard Marconi
Tanguy Marcotty
Todd Margolis
Daniel Marinho
Wanda Markotter
Michael Marks
Venance Maro
Michele Maroli
Paulo Marotto
Mauro Marrelli
Thomas Marrie
Laurent Marsollier
Diana Martin
Coralie Martin
Richard Martin
Julio Martin
Fred Martineau
Beatriz Martinez
Karina Martinez-Mayorga
Elizabeth Martins
Ademir Martins
Francisco Rogerlândio
Martins-Melo
Pascal Mäser
Daniel Masiga
Alessandro Massolo
Cesare Massone
Anuja Mathew
Derrick Mathias
Els Mathieu
Alexander Mathis
Greg Matlashewski
James Matsunaga
Masanori Matsuoka
Keith Matthews
Michael Matunis
Ian Maudlin
Aaron Maule

Isabel Mauricio
Wendy Maury
Alfredo Mayor
Bongani Mayosi
Humphrey Mazigo
Evaristus Mbanefo
Leonard Mboera
Deborah Mc Farland
Monica McArthur
Alan McBride
Christina McCarthy
James McCarthy
Malcolm McConville
Richard McCulloch
Thomas McDonald
Emily McDonald
Mary Ann McDowell
Anita McElroy
Christine Mcgrath
Elizabeth McGraw
Bradford McGwire
Derek McKay
Diane McMahon-Pratt
Don McManus
W. Robert McMaster
Paul McMenamin
Samantha McNulty
Henry McSorley
Jan Mead
Luke Mease
Danielle Medek
Oleg Mediannikov
Freddy Medina
Jane Megid
Terri Meinking
Rojelio Mejia
Peter Melby
Alexander Mellmann
Wayne Melrose
Martin Meltzer
Didier Menard
Tiago Mendes
Rinaldo Mendes
Fela Mendlovic
Clara Menendez
Martin Mengel
Rubem Menna-Barreto
Joris Menten
Margaret Mentink-Kane
Richard Merritt
Pascal Mertens
John Scott Meschke
François-Xavier Meslin
Sharon Messenger
William Messer
Jane Messina
Shulamit Michaeli
Michelle Michalski
Virginie Mick
Christian Miculka
Andrei Mihalca
Steve Mihok
Armin Mikler
Michael Miles
Adrian Miller
Genevieve Milon
Michael Minnick
Sebastián Minoli
Paola Minoprio
Eric Mintz
Democrito Miranda-Filho
Bijay Mirdha
Chad Mire
Guaddalupe Miro
Nerges Mistry
Kate Mitchell
Kendra Mitchell-Foster
Oriol Mitjà
Edward Mitre
Makedonka Mitreva
Hans-Willi Mittrücker
Farrokh Modabber
Christine Moe
Nicolas Moiroux
Igor Mokrousov
Michal Mokry
Israel Molina
David Molyneux
Andrea Mombelli
Hooman Momen
Dinesh Mondal
Karina Mondragon-Shem
Juthathip Mongkolsapaya
Wuelton Monteiro
Martin Montes
Joel Montgomery
Susan Montgomery
Antonio Montresor
Susan Moore
Peter Moore
Sarah Moore
Milton Moraes
Miguel Morales
Serge Morand
Alessandra Morassutti
Diogo Moreira
Luciano Moreira
Otacilio Moreira
David Morens
Jessica Morgan
Lisa Morici
Cory Morin
Ignacio Moriyon
Susan Morpeth
Russell Morphew
James Morris
Christopher Morris
J. Glenn Morris Jr.
Thomas Morrison
W. Ivan Morrison
Amy Morrison
Renato Mortara
Lydia Mosi
Vanessa Mosqueira
Bernard Moss
Eric Mossel
Jeremy Mottram
Adrian Mountford
Ana M. Moura-Da-Silva
Marina Mourão
Marina Mourão
Sara Moutailler
Charles Mowbray
Andreas Mueller
Ivo Mueller
Ivo Mueller
Arup Mukherjee
Rupa Mukhopadhyay
Grace Mulcahy
Albert Mulenga
Pie Müller
Jason Mulvenna
Kosta Mumcuoglu
John Mumford

Hetron Munang'Andu
Ulrike Munderloh
Jose Munoz
Claudia Munoz-Zanzi
Carol Munro
George Munson
Ezekiel Mupere
Grace Murilla
Edward Murphy
Susan Murray
Gerald L Murray
Ahmed Musa
John-Paul Mutebi
Karuppiah Muthumani
Ankur Mutreja
Odwell Muzari
Mercy Mvundura
Jonathan Mwangi
Pauline Mwinzi
Peter Myler
Kevin Myles
Ben Naafs
Ben Naafs
Steven Nadler
Fnu Nagajyothi
Thirumeni Nagarajan
Rana Nagarkatti
Shan Naidoo
Minoru Nakao
Helder Nakaya
Jarlath Nally
Elizabeth Namukwaya
Bakela Nare
Eduardo Nascimento
Ana Nascimento
Theodore Nash
Kalimuthusamy
Natarajaseenivasan
Norman Nausch
Miguel Navarro
Robert Naviaux
Yukifumi Nawa
Momar Ndao
Martial Ndeffo Mbah
Daniel Neafsey
Kenrad Nelson
Nicole Nemeth
Susana Nery
Ana Gisele Neves-Ferreira
Paul Newton
Chiu Ng
Chiu-Wan Ng
Lisa Ng
Lee-Ching Ng
Jeremiah Ngondi
Maina Ngotho
Helena Ngowi
Vinh-Kim Nguyen
William Nicholson
André Nicola
Ana Nicola
Matthias Niedrig
Kirsten Nielsen
Birgit Nikolay
Roshan Niloofa
Leonardo Nimrichter
Alfred K. Njamnshi
Doris Njomo
Joo Hwan No
Flavio Nobre
Harald Noedl
Rita Maria Nogueira
Gregory Noland
Justin Nono
Rahmah Noordin
Steven Norris
Robert Norton
Pierre Nouvellet
Oscar Noya
Belkisyole Noya
Harry Noyes
Tomoyoshi Nozaki
Issarang Nuchprayoon
Márcio Nunes
Thomas Nutman
Patricia Nuttall
Nuha Nuwayri-Salti
Susanne Nylen
Daniel O'Brien
David O'Callaghan
Maria Teresa Ochoa
Torsten Ochsenreiter
Peter Odermatt
Maurice Odiere
Steven O'Hara
Nobuo Ohta
Kazunori Oishi
Joseph Okeibunor
Ana Oleaga
Clelia Oliva
Guilherme Oliveira
Maria Regina Oliveira
Fabiano Oliveira
Sandra Olkowski
Annette Olsen
Peter Olson
Ken Olson
Anders Omsland
Seth O'Neal
Sandra O'Neill
Eng Eong Ooi
Kenneth Opara
Javier Ortego
Omran Osman
Antonio Osuna
Jose Oteo
Rafael Otero
Domenico Otranto
Melanie Ott
Eric Ottesen
Johnson Ouma
Paul Overgaauw
John Overington
Yusuf Ozbel
Krijn Paaijmans
Anne-Laure Page
Frederic Pages
Savita Pahwa
Vivek Pai
Mark Paine
Mariya Pakharukova
Chiranjib Pal
Subhamoy Pal
Pietro Pala
Mark Pallen
Bo Pang
Anna Papa
F. Nina Papavasiliou
Gláucia Paranhos-Baccalà
Alexander Paredez
Daniel Paris
Sang Won Parker
Scott Parker
Andrew Parker

Philippe Parola
Christopher Parry
Nikhat Parveen
Parviz Parvizi
A. Lorena Passarelli
Arvind Patel
Milind Patole
Fabiana Paula
Christophe Paupy
Christopher Peacock
Mark Pearson
Terry Pearson
Joao Pedra
Jorge Pedrosa
Rosanna Peeling
Mark Peeples
Raymond Peirce
Tao Peng
Pamela Pennington
Claudio Pereira
Marcos Pereira
Marcia Pereira De Oliveira
Vera Lucia Pereira-Chioccola
Mercio Pereiraperrin
Rushika Perera
Esther Perez
Carmen Perez-Guerra
Alex Perkins
Robert Perry
Christine Petersen
Brett Petersen
Jennifer Peterson
Townsend Peterson
William Petri, Jr.
Christophe Peyrefitte
Kenneth Pfarr
Edward Pfeiler
Minh Duy Phan
Christina Phares
Margaret Phillips
Richard Phillips
Renaud Piarroux
Mathieu Picardeau
Amy Pickering
Raymond Pierce
Ted Pierson
Joseph Piesman
David Pigott
Gorben Pijlman
Tom Pike
Davita Pillay
Allan Pillay
Phileno Pinge-Filho
Rejane Pinheiro
Daniel Pinschewer
Eduardo Pinto
Claude Pirmez
R. Pitts
Virginia Pitzer
Juan Pizarro
Raina Plowright
Gerd Pluschke
Larissa Podust
Anne Poinsignon
Rafael Polidoro
Michael Pollastri
Anastasia Polycarpou
Christelle Pomares
Marco Pombi
Alongkot Ponlawat
Alicia Ponte Sucre
Rory Post
Erik Post
Miriam Postan
Rodolphe Poupardin
Michael Povelones
Jeffrey Powell
Ann Powers
Edoardo Pozio
Clarissa Prazeres Da Costa
Ric Price
Dana Price
Victor Prieto
Anna Protasio
Narain Punjabi
Rituraj Purohit
Alyssa Pyke
Farah Qamar
Flavio Queiroz Telles
Rupert Quinnell
Jorge Rabinovich
Sima Rafati
Ram Raghavan
Vedantam Rajshekhar
Stuart Ralph
Kapa Ramaiah
Marcel Ramirez
Juan David Ramirez
Grant Ramm
Angel Ramos-Ligonio
Alice Ramyil
Elizabeth Rangel
Hilary Ranson
Francis Raoul
Chad Rappleye
Rino Rappuoli
Jason Rasgon
David Rasko
David Rasmussen
Debalina Ray
Jean Baptiste Rayaisse
Julian Rayner
Laurie K Read
Paul Ready
Eduardo Rebollar
Sergio Recuenco
Anand Reddi
Steven Reed
Will Reeves
Ryan Rego
Michael Reich
Simon Reid
Daniel Reidpath
Juliana Reimão
Lisa Reimer
Robert Reiner
William Reisen
Paul Reiter
Richard Reithinger
Jan Remme
Juan Rendon
Alfons Renz
Ana Requena-Méndez
Stephen Resch
Serge Resnikoff
Olivier Restif
Guilherme Ribeiro
Paula Ribeiro
José Ribeiro
Paulo Ribeiro Jr
Paulo Ribolla
Natasha Ricci
Frank Richards
S. Richards

Rebecca Richards-Kortum
Joshua Richardson
Joachim Richter
Rebecca Rico-Hesse
Suman Rijal
Sébastien Rioux Paquette
Scott Ritchie
Luis Rivas
Jacob Riveron
Carlos Robello
Kis Robertson
Mark Robinson
Prema Robinson
Luis Rocha
Manoel Otávio Rocha
Benjamin Roche
Louise Rodino-Klapac
Mauricio Rodrigues
Marcio Rodrigues
Nilton Rodrigues
Virmondes Rodrigues Jr
Ana Rodriguez
Alexis Rodriguez-Acosta
Isabel Rodriguez-Barraquer
Dawn Roellig
Ester Roffe
Michael Rogan
Matthew Rogers
Matthew Rogers
Stephen Rogerson
Pejman Rohani
Iva Rohousova
David Roiz
Antonieta Rojas De Arias
Gustavo Romero
Claudia Romero-Vivas
Thomas Romig
Phyllis Romijn
Pierre Roques
Helene Rosenberg
Mara Cecilia Rosenzvit
Paul Rosile
Alan Rothman
Brice Rotureau
Lubka Roumenina
Francoise Routier
Scott Roy
Syamal Roy
Matthew Rubach
Larry Ruben
Guita Rubinsky-Elefant
Robert Rudd
Gloria Rudenko
Vurayai Ruhanya
Diego Ruiz-Moreno
Andreas Ruppel
Bruce Russell
Terence Ryan
Edward Ryan
Gustaf Rydell
Juliana Sá
Christopher Saarnak
Karla Saavedra-Rodriguez
David Sack
Moussa Sacko
David Sacks
Bibhuti Saha
Bhaskar Saha
Masayuki Saijo
Judy Sakanari
Naseem Salahuddin
Marcel Salathé
Pablo Salazar
Juan Salazar
Claudio Salgado
Mustafa Salih
Jorge Salinas
Henrik Salje
Jeanne Salje
Oscar Salomon
Francisco Salzano
Indira Samarawickrema
Maganga Sambo
Ahmed Samir
Richard Samuels
John Samuelson
Jose Sanchez
Ana Sanchez
Lucas Sánchez
Johan Sandberg
Jaime Santana
Mauricio Sant'Anna
Helton Santiago
Marcelo Santoro
Renato Santos
Reinaldo Santos
Vanessa Sarathy
Fred Sarfo
Rajiv Sarkar
Adriana Sarmento
Euzenir Sarno
Ana M. Sartori
Sachiko Sato
Jetsumon Sattabongkot
Paul Saunderson
Harry Savage
Hirofumi Sawa
Kittisak Sawanyawisuth
Somphou Sayasone
Donald Schaffner
Julio Scharfstein
Pauline Scheelbeek
Sergio Schenkman
John Schieffelin
Alejandro Schijman
Connie Schmaljohn
Wolf-Peter Schmidt
Jonas Schmidt-Chanasit
Erich Schmutzhard
Achim Schnaufer
Jay Schneider
Maria Cristina Schneider
Barbara Schnierle
Randal Schoepp
Steve Scholand
Claus-Jürgen Scholz
Gabriele Schönian
Wolfgang Schulz
Claudia Schulz
Tom Schwan
Ira Schwartz
Eli Schwartz
Alexandra Schwarz
Herbert Schweizer
Edda Sciutto
D.M. Scollard
Kézia Scopel
Diana Scorpio
Thomas Scott
William Secor
Nágila Secundino
Anthony Seddoh
Antonio Seguro
Karin Seifert

Zusana Sekeyová
Angamuthu Selvapandiyan
Paul Selzer
Roshanak Semnani
Esteban Serra
David Serre
Alexandre Servat
October Sessions
Anaiá Sevá
Carlo Severini
David Severson
Sean Shadomy
G. Dennis Shanks
Michal Shapira
Tyler Sharp
Alex Shaw
Thomas Shelite
Donald Shepard
Pei-Yong Shi
Jinjun Shi
Pei-Yong Shi
Clive Shiff
Maria Shikanai-Yasuda
Jyh-Wei Shin
Tatsuo Shioda
Sujan Shresta
Ambuj Shrivastava
Stephanie Shwiff
Hanna Sidjabat
Ariel Silber
Sydnei Silva
João Silva
Mario Silva-Neto
Cameron Simmons
Fabrice Simon
Mark Simons
Paul Simonsen
Sami Simsek
Calvin Sindato
Steven Singer
Brajendra Singh
Balbir Singh
Sarman Singh
Shailza Singh
Neeloo Singh
Gagandeep Singh
André Siqueira
Jair Siqueira-Neto
Amir Siraj
Chukiat Sirivichayakul
Mark Sistrom
Paiboon Sithithaworn
Nopporn Sittisombut
Martina Siwek
Lenka Skálová
Patrick Skelly
Marvin Sklar
Barton Slatko
Mark Slifka
Michel Slotman
David Šmajs
Pamela Small
Timo Smieszek
Pablo Smircich
Darci Smith
Deborah Smith
Terry Smith
Helen Smith
Lucy Smith Paintain
Milena Soares
Rodrigo Soares
Philippe Solano
Laia Solano-Gallego
Hoorieh Soleimanjahi
Anthony Solomon
Yajun Song
Lynn Soong
Ghislain Sopoh
Sergio Sosa-Estani
Javier Sotillo
Esteban Soto
Paul Southern
Luis Carlos De Souza Ferreira
Robert Spear
Sabine Specht
Benjamin Speich
Dave Speijer
John Spencer
Niko Speybroeck
Markus Spiliotis
Cynthia Spillmann
Terry Spithill
David Spray
Amy Springer
Anon Srikiatkhachorn
Ashley St John
Colin Stack
Simona Stäger
Lola Stamm
Jeffrey Stanaway
Miles Stanford
Michelle Stanton
J. Erin Staples
Peter Steinmann
Ivo Steinmetz
John Stenos
Nicole Stephenson
Brian Stevenson
Philip Stewart
Karin Stiasny
Ymkje Stienstra
Benoit Stijlemans
Dan Stinchcomb
Timothy Stinear
Robyn Stoddard
Marija Stojkovic
Wilma Stolk
J. Russell Stothard
Mary Straub
Adrian Streit
Claudio Struchiner
Richard Strugnell
Stavana Strutz
Chunlei Su
Brian Suarez Mantilla
Jonathan Sugimoto
Andreas Suhrbier
John Sullivan
Nancy Sullivan
David Sullivan, Jr.
Peifang Sun
Sujai Suneetha
Taniawati Supali
Frantisek Supek
Mehul Suthar
Laura Sutherland
Sutas Suttiprapa
Koichi Suzuki
Staffan Svard
Gote Swedberg
Paul Swoboda
Din Syafruddin
Zainulabeuddin Syed
Walter Tabachnickk

Carlos P. Taborda
Hiroshi Tachibana
Fabienne Tachini-Cottier
Howard Takiff
Kawsar Talaat
Benoit Talbot
Fasihah Taleo
Sinesio Talhari
Carolina Talhari
Francesca Tamarozzi
Paul Tambyah
Nget Hong Tan
Choo Hock Tan
Babasaheb Tandale
Qing Tang
Fréderic Tangy
Steven Tannenbaum
Herbert Tanowitz
Dingyin Tao
Wagner Tassinari
Mark Taylor
Martin Taylor
Steve Taylor
Fabrizio Tediosi
Antonio Teixeira
Antonio Teixeira
Marcus Teixeira
Maria Glória Teixeira
Andréa Teixeira-Carvalho
Andrea Teixeira-Carvalho
Sandra Telfer
Sam Telford Iii
Sharon Tennant
Jacob Tennessen
Andrew Teo
Luis Terrazas
Robert Terry
Robert Tesh
Saravanan Thangamani
Marc Thellier
Alejandro Thiermann
Stephen Thomas
Lian Thomas
Matthew Thomas
Ricardo Thompson
Richard Christopher
Thompson
Graham Thornicroft
Guy Thwaites
Huai-Yu Tian
Natalia Tiberti
Kirkby Tickell
Leopold Tientcheu Djomkam
Kit Tilly
Peter Tinnemann
Daniel Tisch
Richard Titball
Adriane Todeschini
Max Jean Toledo
Kay Tomashek
Noel Tordo
Paul Torgerson
Paul Torgerson
Steve Torr
Jaime Torres
Alfredo Torres
José Tort
Pablo Tortosa
Chafia Touil-Boukoffa
Rebecca Traub
Lydie Trautmann
Bruno Travi
Robert Trengove
Omar Triana-Chávez
Richard Truman
James Truscott
Anastasios Tsaousis
Rea Tschopp
Fan-Chen Tseng
Konstantin Tsetsarkin
Renée Tsolis
Apichai Tuanyok
Suely Tuboi
James Tumwine
Michael Turell
Hugo Turner
Joseph Turner
Hugo Turner
Kevin Tyler
Florencio Ubeira
Christopher Uejio
Uade Ugbomoiko
Silvia Uliana
Buddy Ullman
Eduardo Undurraga
Britta Urban
Michael Urbaniak
Julio Urbina
Jürg Utzinger
Jude Uzonna
Pierrick Uzureau
Sakthivel Vaiyapuri
Gustavo Valbuena
Glyn Vale
Olaf Valverde Mordt
Govert van Dam
Cornelia van de Weg
Jan Van Den Abbeele
Andrew van den Hurk
Gert van der Auwera
Barbara Van Der Pol
Adrianus van der Velden
Tjip van der Werf
H. Rogier van Doorn
Johan van Griensven
Lisette van Lieshout
Wesley Van Voorhis
Ger van Zandbergen
Manu Vanaerschot
Katrien Vanbocxlaer
Koen Vandelannoote
Dieter Vanderelst
Luc Vanhamme
Veerle Vanlerberghe
Pedro Vasconcelos
Nikos Vasilakis
Jefferson Vaughan
Muriel Vayssier-Taussat
Susana Vaz Nery
Gonzalo Vazquez-Prokopec
Ivan Velez
Laia Ventura-Garcia
Stefano Veraldi
Patricia Veras
Manuela Verastegui
Anibal Vercesi
Baptiste Vergnes
Alan Verkman
Sten Vermund
Jaco Verweij
Diego Viasus
Paulo Vieira
Marco Vigilato
Stalin Vilcarromero

Fernando Villalta
Sharon Villanueva
Luis Villar
Luis Eduardo Martinez Villegas
Joseph Vinetz
Rodolfo Viotti
Johnny Vlaminck
Petr Volf
Veronika von Messling
Lorenz von Seidlein
Esther von Stebut
Sirenda Vong
John Vontas
H. Martin Vordermeier
Jan Votypka
Dominique Vuitton
Wolfgang Wüster
Timothy Wade
Jesse Waggoner
Abdul Waheed
Helene Wahlström
Matthew Waldor
Etienne Waleckx
Henry Walke
Martin Walker
Edward Walker
Stephen Walker
David Walker
Ryan Wallace
John Wallace
Lance Waller
L.A. Waller
Pegine Walrad
Douglas Walsh
Judd Walson
Catherine Walton
Linda Wammes
Shih-Min Wang
Baoxi Wang
Tian Wang
Wei-Kung Wang
Wei Wang
Mark Wansborough-Jones
Jonathan Warawa
Alon Warburg
Honorine Ward
Nicola Wardrop
Kelly Warfield
Jeffrey Warner
David Warrilow
James Wasmuth
Gideon Wasserberg
Steve Waterman
Alice Wattam
Douglas Watts
Matthew Watts
Scott Weaver
Emily Webb
Bonnie Webster
Gareth Weedall
Mirani Weerasooriya
David Weetman
Gary Weil
Tiffany Weinkopff
Philip Weinstein
Daniela Weiskopf
Brian Weiss
Melanie Wellington
Oliverio Welsh
Hao Wen
Thomas Wenzel
Karl Werbovetz
Luciana Wermelinger
Guilherme Werneck
Catherine Werts
T. Eoin West
Sheila West
Adrian Whatmore
Lisa White
Julian White
Bradley White
Laura White
A. White, Jr.
Dominic Wichmann
Stefan Wiehr
W. Wiersinga
Henry Wilde
Annelies Wilder-Smith
Patricia Wilkins
Annie Wilkinson
Roderick Williams
David Williams
Craig Williams
Brian Williams
Diane Williamson
Heather Williamson-Jordan
Arve Willingham
Rodney Willoughby
Bridget Wills
Shona Wilson
R. Alan Wilson
William Wilson
Mary Wilson
Ken Winkel
Andrea Winkler
Michael Witty
Laila Woc Colburn
Dawit Wolday
Adrian Wolstenholme
Kimberly Won
Charles Wondji
Pak Kin Wong
Surasakdi Wongratanacheewin
Christopher Woods
Alistair Woodward
Mark Woolhouse
Henry Wortis
Zhiliang Wu
Reinhard Wuerzner
Betty Wu-Hsieh
Elsio Wunder Jr
Vanaporn Wuthiekanun
Sarah Wynwood
Chuanwu Xi
Liyan Xi
Zhiyong Xi
Jianguo Xia
Shang Xia
Zhiquan Xiang
Lihua Xiao
Cecilia Ximenez
Xiaolu Xiong
Chaoyang Xue
Kazunori Yamaguchi
Hiroshi Yamasaki
Gavin Yamey
Jie Yan
Tetsuya Yanagida
Ruifu Yang
Gyongseon Yang
Wan Yang
S. Yanow

Cedric Yansouni
Peiling Yap
Dorothy Yeboah-Manu
Janet Yee
Trai-Ming Yeh
Jonathan Yoder
Lisa Yon
In-Kyu Yoon
Nobuko Yoshida
Timothy Yoshino
Hong You
Neil Young
Megan Young
Paul Young
Laura Youngblood
Xue-Jie Yu
Paolo Zanotto
Dante Zarlenga
Fidel Zavala
Jim Zeegelaar
Herve Zeller
Jonathan Zelner
Peibin Zeng
Bin Zhan
Yaobi Zhang
Si-Ming Zhang
Kai Zhang
Qin Zhao
Xian-Zheng Zhou
Eduard Zijlstra
Dan Zilberstein
Bianca Zingales
Jakob Zinsstag
Matt Zinter
Carina Zittra
Wolfram Zueckert
Rimma Zurabian
Marcel Zwahlen
Ellen Zwarthoff
